# Utilization of Several Industrial Wastes as Raw Material for Calcium Sulfoaluminate Cement

**DOI:** 10.3390/ma12203319

**Published:** 2019-10-12

**Authors:** Phongthorn Julphunthong, Panuwat Joyklad

**Affiliations:** 1 Department of Civil Engineering, Faculty of Engineering, Naresuan University, Phitsanulok 65000, Thailand; 2 Department of Civil and Environmental Engineering, Faculty of Engineering, Srinakharinwirot University, Nakhon Nayok 26120, Thailand

**Keywords:** calcium sulfoaluminate cement, industrial wastes, ye’elimite, Rietveld refinement technique, compressive strength

## Abstract

The aim of this research was to study the production of calcium sulfoaluminate (CSA) cement from several industrial waste materials including with marble dust waste, flue gas desulfurization gypsum, ceramics dust waste, and napier grass ash. The chemical composition, microstructure, and phase composition of raw materials were examined using energy dispersive X-ray fluorescence (EDXRF), scanning electron microscopy (SEM), and X-ray diffraction (XRD), respectively. All raw wastes were analyzed using their chemical composition to assign proportion for raw mixture. The raw mixture is calcined at controlled calcination temperatures ranging from 1200 °C to 1300 °C for 30 min. Subsequently, with analysis, their phase composition is calculated by the Rietveld refinement technique. The results suggested that phase composition of clinker calcined at 1250 °C shows the closest composition when compared to target phases, and was selected to prepare CSA cement. The FTIR analysis was performed to study the hydration processes of CSA cement. The Ordinary Portland cement (OPC) based with adding CSA cement between 20 wt.% and 40 wt.% were investigated for the effect of CSA cement fraction on water requirement, setting times and compressive strength. The results showed that rapid setting and high early strength can be achieved by the addition of 20–40 wt.% CSA cement to OPC.

## 1. Introduction

The utilization of by-products or industrial wastes as additions to Ordinary Portland Cement (OPC) is a well-known technique to reduce the CO_2_ emissions associated with the energy-intensive manufacture of cement [1]. An alternative way to reduce the embodied CO_2_ (eCO_2_) from OPC production is the use of a non-PC based system as a binding ingredient. Calcium sulfoaluminate cement (CSA cement) is one such alternative binder which lowers eCO_2_ emission compared to OPC. CSA cement was originally developed in China in the 1970s and has been used since then [2]. Gartner [3] studied the eCO_2_ emissions of individual cement components, and estimated that the eCO_2_ emission from producing a typical CSA cement consisting of ye’elimite, belite and, aluminoferrite is approximately 600 kg/t. This represents an eCO_2_ reduction of approximately 35% compared to OPC [1]. The total reduction of eCO_2_ emission is the result of three main causes: Reduction of burning temperature by approximately 100–150 °C, reduction of energy consumption during the grinding process due to easier grindability, and reduction of eCO_2_ due to a lower fraction of CaCO_3_ in the raw materials.

CSA cements all include ye’elimite (C_4_A_3_$), belite (C_2_S), and aluminoferrite (C_4_AF) phases, which are in different proportions depending on the application [4,5,6]. CSA cements have demonstrated rapid setting, high early-age strength, self-stressing, and shrinkage compensating properties due to the fast reacting C_4_A_3_$ and the expansive nature of ettringite [7,8,9,10,11,12,13,14,15,16,17,18,19]. In field practices, CSA cements have been used mainly in pre-cast concrete applications and repair applications due to their high early-age strength development. However, industrial scale production and usage of CSA cements is still limited in China [20]. The main factors holding back widespread production of CSA cement is its relatively high cost and the limited availability of the required raw materials. Natural deposits of calcium and silicon oxides are plentiful throughout most of the world, making it possible to produce Ordinary Portland cement cheaply in most areas using local materials. However, CSA cements produced for commercial use contain large amounts of C_4_A_3_$, which requires a higher amount of aluminum oxide than in Ordinary Portland cement. The most commonly used source of aluminum is bauxite deposits which are not widespread. Additionally, the extracted alumina can be expensive.

Thailand has begun to transform its economy from agriculture to export-oriented manufacturing, while integrating key manufacturing production into the regional value chain. This transformation has led to the production of various types of industrial wastes in large amounts, which inevitably become an environmental problem. In this study, a variety of industrial waste materials including marble dust waste, flue gas desulfurization gypsum, ceramics dust waste, and napier grass ash were selected to use as raw materials in the synthesis of CSA cement. These raw ingredients were proportioned according to the phase composition calculated from a modified Bogue’s equation. The finished CSA cements synthesized from waste materials were then further studied to verify their actual phase composition and hydration processes. Finally, Ordinary Portland cement–CSA cement blends with various proportions were investigated in order to determine the influences of CSA cement content on water requirement, setting time, and compressive strength of the cement paste. 

## 2. Materials and Methods 

### 2.1. Raw Materials and Sample Preparation for CSA Clinker

The industrial waste materials selected for synthesis of the CSA clinker were provided from several sources. Marble dust waste (MDW) was provided in the form of a wet slurry from a marble factory in Kampheang Phet Province, Thailand. Flue gas desulfurized gypsum (FGDG) was procured from Mae Moh Power Plant in Lampang Province, Thailand. Ceramic dust waste (CDW), which is produced during the final polishing process of ceramic tiles, was supplied from Ceramic Chemical Refractory, Lampang Province, Thailand. Napier grass ash (NGA) was collected from Vithai Biopower Plant, Uthai Tanee Province, Thailand. All waste materials were dried in an oven at 80 °C for 24 h and then sieved through 150 µm mesh. The mineralogical compositions and microstructure of these waste materials were investigated using X-ray diffraction (XRD) and scanning electron microscopy (SEM) images, respectively. Finally, chemical compositions of the four materials were analyzed using energy dispersive X-ray fluorescence (EDXRF). Data on the chemical compositions of the waste materials was used to estimate the proportions of raw materials for the mixture using the modified Bogue’s equation calculation [21] for the targets C_4_A_3_$ (~50 wt.%), C_2_S (~40 wt.%), and C_4_AF (~10 wt.%). Analytical-grade Al_2_O_3_ was added as a raw material (25 wt.%) to obtain the desired composition. CaF_2_ was added to the starting materials (0.8 wt.%) to accelerate and enhance their reactivity [22].

The calculated amounts of starting materials were weighed and mixed in a ball-mill with ethanol for 24 h. The suspension was dried using a hot plate, ground using an agate mortar, and then sieved into a fine powder. The powder was molded into spherical samples (~10 g and 10 mm diameter), and the samples were placed in Pt/Rh crucibles and fired in an electric furnace. The firing process consisted of two steps. In the first step, the samples were calcined at 800 °C for 30 min to dehydrate and calcine the raw ingredients. In the second step, the samples were heated up to three different temperatures (1200 °C, 1250 °C, and 1300 °C, respectively) and fired for 30 min. In all cases the heating rate of the furnace was 5 °C/min. After the second step, the CSA clinker was immediately removed from the furnace and rapidly cooled. 

### 2.2. Analytical Methods 

#### 2.2.1. Energy Dispersive X-ray Fluorescence (EDXRF)

The energy dispersive X-ray fluorescence (EDXRF) analytical technique was employed to determine the chemical composition of the raw materials and synthesized CSA clinker. This investigation utilized the interaction of X-rays with each material to determine each sample’s elemental composition using a Horiba XGT-5200 X-ray analytical microscope.

#### 2.2.2. X-ray Diffraction (XRD)

The XRD analysis was carried out in order to determine the mineralogical phase of the raw materials, the synthesized clinker, and the OPC. The dried samples were then ground into a fine powder and sieved through 150-mesh (opening of 104 µm.). The analysis was performed with a PAnalytical X’pert Pro powder diffractometer with Cu-Kα radiation (1.54187 Å, 40 mA, 40 kV) as the radiation source. The powder patterns were gathered in the 2θ range of 10–60°. Rietveld refinement quantitative phase analyses of the CSA clinker and the OPC were done using DIFFRAC.SUITE TOPAS V.5.0 software. The refined parameters included phase scale factors, background coefficients, zero-shift error, lattice parameters, peak shape parameters, and preferred orientation, if needed. As for reliability of the data, with the software’s “goodness of fit” setting fixed to <4, the fitted curve matched well with the raw data [23,24,25]. 

#### 2.2.3. Scanning Electron Microscopy (SEM)

Scanning electron microscopy (JEOL JSM 5910 LV), was used to observe the morphology and the particle size of the raw materials. The samples were finely grinded into powders with an agate mortar and sieved through the 150 µm-opening mesh. Prepared powders were coated with a thin layer of gold to promote electrical conductivity further to microstructure characterization.

#### 2.2.4. Fourier-transform Infrared Spectroscopy (FTIR)

The Fourier-transform infrared (FTIR) analysis was performed using a Perkin Elmer FTIR System Spectrum X spectrometer in the range of 400–4000 cm^−1^ with spectral resolution of 1 cm^−1^. This analytical technique was used to identify the functional group signals of the anhydrous cement and hydrated pastes. The specimens were ground to a fine powder using a mortar and pestle. For hydrated pastes, the samples were washed with acetone to remove water in an effort to mitigate the hydration reaction. After washing with acetone, the samples were thoroughly dried in an oven at 45 °C for an hour.

#### 2.2.5. Water Requirement, Setting Time and Compressive Strength of Various CSA Blends

Currently, most countries use expanding CSA-based ternary blends consisting of CSA clinker, added gypsum, and OPC [26,27]. The current study also used this combination, based on the novel CSA clinker which was prepared from industrial wastes. In order to test various blends containing CSA clinker, six systems were selected to test the engineering properties of their cement pastes. The first system is 100% OPC, and this system is referred to as OPC. The remaining five systems are all CSA/OPC blends consisting of CSA clinker initially mixed with 20 wt.% of natural gypsum [28] (abbreviated as CSA cement), followed by addition of an amount of OPC to yield a net CSA cement content of 20%, 25%, 30%, 35% and 40% by weight. Thus, the five blended systems are referred to as CSA20, CSA25, CSA30, CSA35, and CSA40, reflecting their CSA cement content by weight (Table 1). The amount of water required by each test system for normal consistency of its cement paste was determined using the ASTM C187 test [29]. The initial and final setting times of the cement pastes were found by performing the ASTM C191 test [30]. For the compressive strength test, each fresh paste was cast into 25 mm acrylic cube molds according to the method of Rungchet et al. [31] with a w/b ratio of 0.34. The specimens were demolded at the age of 3 h and cured in a moisture-controlled room at 20 °C with 95% R.H. Their compressive strength was tested at the curing periods of 6, 12, 24, 72, 168, 336 and 672 h.

## 3. Results and Discussion

### 3.1. Characterization of Raw Materials

**Figure 1:**
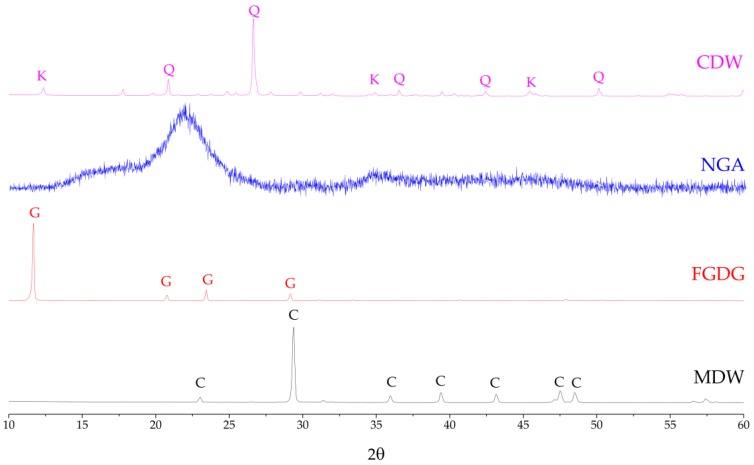
X-ray diffraction patterns of the four waste materials. C = calcite, G = gypsum, Q = quartz, and K = kaolinite.

Figure 1 shows the XRD patterns of the industrial waste materials which were selected as starting materials. The main mineral component of MDW is calcite (CaCO_3_), while that of FGDG is gypsum (CaSO_4_**·**2H_2_O). The XRD patterns of NGA reveal an amorphous phase. CDW has two crystalline phases: Quartz (SiO_2_) and kaolinite (Al_2_Si_2_O_5_(OH)_4_). The results of the EDXRF chemical composition tests agreed with and confirmed the mineralogical analysis results from the XRD patterns, as shown in Table 2.

**Figure 2:**
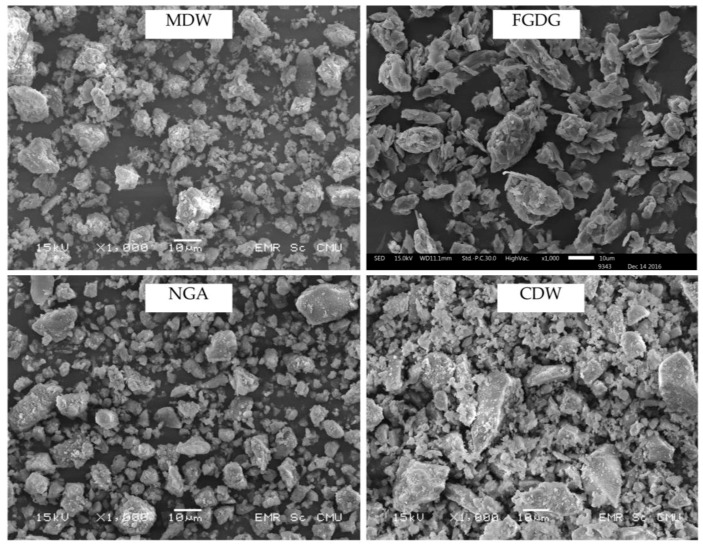
SEM images of the four raw materials used.

The SEM micrographs of MDW, FGDG, NGA and CDW powders are shown in Figure 2. CDW shows an irregular shape and a slight agglomerated form. A well-grade particle size distribution was observed with a range of lower than 1 µm to higher than 20 µm. For FGDG powders, a plate-like shape with medium agglomeration was observed. The agglomerate sizes vary from 10 μm to 30 μm but the majority of them vary in size from 15 μm to 20 μm. For NGA, a porous agglomerated form powders of about 5–15 µm size are seen with close to spherical shapes. CDW powders exhibited an irregular shape with a gap-grade of particle size distribution. They consist of the large particles with irregular shape and small particles with porous agglomerated form. 

### 3.2. Characterization of CSA Clinkers 

The target chemical composition of the raw mixture is shown in Table 2. The clinker which was fired at 1250 °C was chosen for analysis of its chemical composition using the EDXRF technique (Table 2). The chemical composition of the clinker showed slight differences when compared to the target composition due to the inhomogeneity of the waste materials themselves.

**Figure 3:**
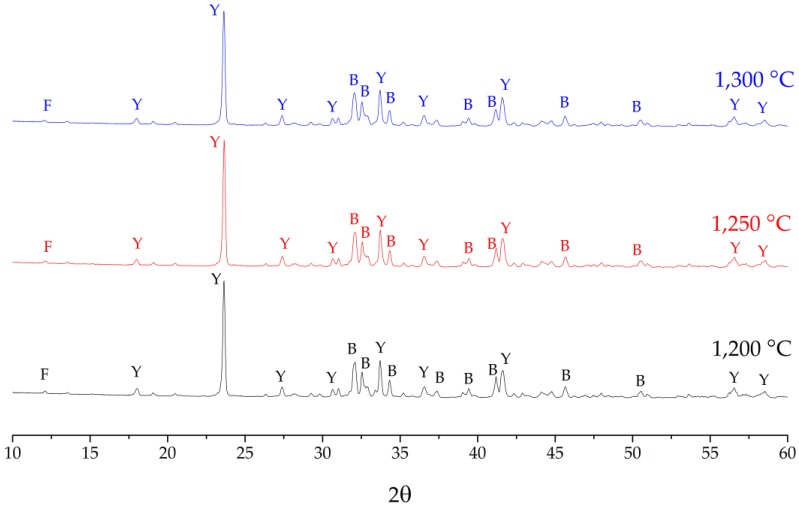
X-ray diffraction patterns of CSA clinkers fired at difference temperatures: Y = ye’elimite, B = belite, F = brownmillerite.

Figure 3 illustrates the XRD patterns of CSA clinkers fired at various temperatures. The main phases in all investigated clinkers are ye’elimite and belite, which is consistent with the target phases. However, the diffraction patterns of samples fired with various temperatures shows a slight difference, and it is difficult to identify the phases of evolution due to the changing firing temperature. Thus, the changes in phase composition due to increase firing temperature were qualified by the Rietveld analysis and the results are shown in Table 3. For clinker fired at 1200 °C, ye’elimite and belite are dominant phases which are consistent with the target composition. The phase content of ye’elimite and brownmillerite are slightly lower than that of the target phase content while belite is slightly higher. A small amount of unassigned fraction, mayenite and bassanite, was found. By increasing the firing temperature to 1250 °C, the phase content of ye’elimite, belite and brownmillerite was changed and more closely reached the target composition with decreasing of the unassigned fraction. However, an increase of firing temperature to 1300 °C caused the synthesized clinker showing more difference from the target composition. The firing condition at 1250 °C for 30 min was selected to the prepared sample to study the hydration processes.

### 3.3. Characterization of Hydrated Paste

The clinker was ground and then sieved through 150 µm mesh, and subsequently mixed with natural gypsum (CaSO_4_**·**2H_2_O) at a weight ratio of 80:20 to obtain CSA cement. This calcium sulfate (CaSO_4_**·**2H_2_O) content can be transferred into a calcium sulfate to ye’elimite molar ratio (value M) of 1.5. According to a formula for calculating the optimum sulfate level for calcium sulfoaluminate cement, a value M between 0 and 1.5 yields rapid hardening and high strength properties [32,33]. When CSA cement is mixed with water, ye’elimite reacts quickly with calcium sulfate and results in the formation of ettringite (C_6_A$_3_H_32_) and aluminum hydroxide (AH_3_) as shown in Equation (1) [27]. The formation of ettringite fills the available space rapidly, providing the paste with good early-age strength [32].

When the calcium sulfate is fully depleted, the hydration of ye’elimite continues with the formation of monosulfate (C_4_A$H_12_) [28], as seen in Equation (2). Theses reactions usually start forming during the age range from 6 h to 48 h, and the formations of ettringite and monosulfate are strongly influenced by the type and dosage level of calcium sulfate [34,35]. Compared with ye’elimite, belite hydrates more slowly. Belite reacts with free water to form calcium silicate hydrates (C–S–H) and portlandite (Ca(OH)_2_) as shown in Equation (3). However, the simultaneously hydration reactions of ye’elimite, belite and brownmillerite caused complex reactions to occur. Belite may react with aluminum hydroxide, that is formed by the hydration of ye’elimite, and the main crystalline product is strätlingite (C_2_A$H_8_), as seen in Equation (4), and may be continuously reacting to form hydrogarnet (C_3_A$_x_H_6-2x_), as seen in Equation (5) [32]. On the other hand, calcium hydroxide, mainly generated from the hydration of belite, may be combined with ye’elimite and gypsum, and also generated expansive ettringite as shown in Equation (6) [34].

(1) $$C_{4}A_{3}\$\;+\;2C\$\;+\;38H\to C_{6}{A\$H}_{32}+2{\mathrm{AH}}_{3}$$

(2) $$C_{4}A_{3}\$\;+\;18H\to C_{4}A\$H_{12}+2AH_{3}$$

(3) $$C_{2}S\;+\;4.3H\to C_{1.7}SH_{4}+0.3CH$$

(4) $$C_{2}S\;+\;AH_{3}+5H\to C_{2}A\$H_{8}$$

(5) $$9C_{2}S\;+\;C_{2}A\$H_{8}\to 10C_{1.7}\$H_{4}+C_{3}AH_{6}$$

(6) $$C_{4}A_{3}\$\;+\;6CH+8C\$\;+\;90H\to 3C_{6}A\$H_{32}$$

**Figure 4:**
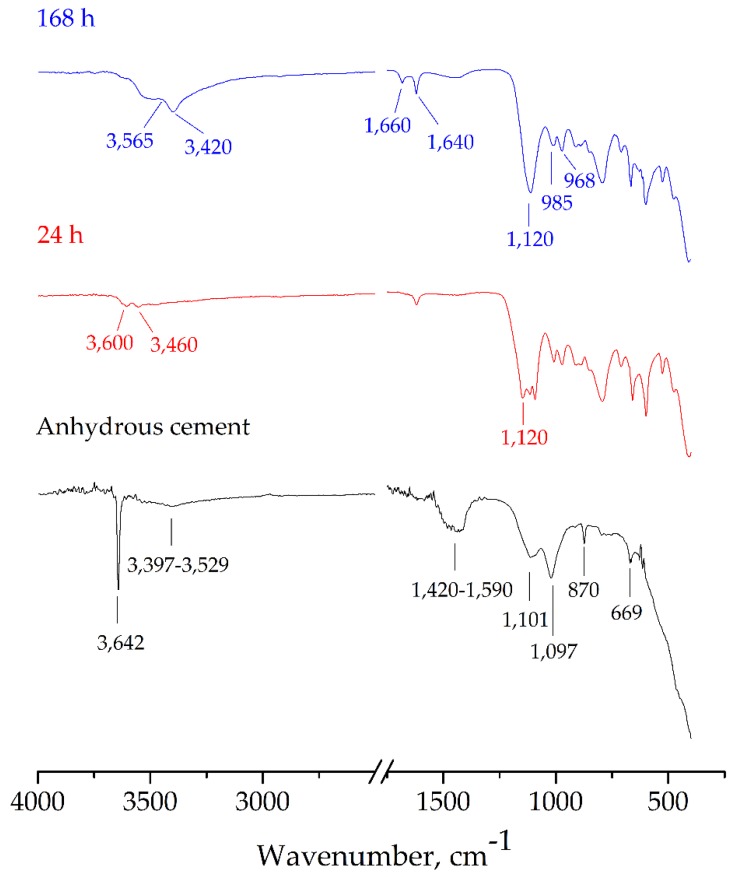
FTIR spectrum of anhydrous cement and hydrated pastes cured for 24 h and 168 h.

Figure 4 shows the FTIR spectra of CSA cement and hydrated pastes at several hydration ages. For anhydrous cement, ye’elimite can be identified by two typical regions. The first one is the ν_3_SO^−2^_4_ group vibration peaks centered in 1097 and 1101 cm^−1^; the second one in the adsorption region, 725–788 cm^−1^, is interpreted as stretching vibrations of a lattice of AlO_4_ tetrahedra [36]. The double peak at 870 and 940 cm^−1^, assigned to Si–O symmetric and antisymmetric stretching of Si-O bonds within tetrahedral SiO_4_ groups, characterizes mainly C_2_S phase [37,38,39]. C_4_AF phase cannot be identified by the FTIR technique due to poorly resolved bands and very small amount. The small peaks at 601 and 669 cm^−1^ are assigned to the bending modes of sulfate in gypsum. The stretching vibrations of the H_2_O molecules in the gypsum identified by the broad band in the range of 3397–3529 cm^−1^. The unique peak at 3642 cm^−1^ and a broad adsorption band site between 1420 and 1590 cm^−1^ were observed due to the presence of O–H stretching and vibration modes C–O of carbonate groups, respectively [38]. This suggested portlandite impurity was found and may be caused from natural gypsum contamination. The FTIR spectra of paste hydrated for one day presented a very strong anti-symmetrical centred towards 1120 cm^−1^, and can be attributed to stretching frequency of ν_3_SO^−2^_4_ group of ettringite [39]. When considered with the disappearance of a strong peak at 3642 cm^−1^ due to portlandite, and a broad band in the range of 3397–3529 cm^−1^ due to gypsum, this suggested that the hydration reaction of ye’elimite following Equation (6) probably occurred. Moreover, an appearance of two small sharp bands with area between 3500 and 3600 cm^−1^ involves to C_3_AH_6_, AH_3_ and C_2_A$H_8_, which indicated the hydration reaction of C_2_S according to Equations (4) and (5) probably occurred [36]. After curing for 7 days, the FTIR spectra showed three distinct adsorption bands of ettringite and a very strong anti-symmetrical stretching frequency of the sulphate ion (ν_3_SO^−2^_4_) centred towards 1120 cm^−1^. The water absorption band appeared at 1640 cm^−1^ for ν_2_H_2_O and a broad band at 3420 and 3635 cm^−1^ due to ν_1_H_2_O and νOH_free_, respectively [39]. A medium sharp at 1660 cm^−1^ that was assigned to O–H_capillary_ stretching vibrations and two sharp bands at 963 and 985 cm^−1^ due to Si–O stretching vibrations of C_1.7_SH_4_. These observations suggested the hydration reaction of ye’elimite and belite.

### 3.4. Engineering Properties of OPC-CSA Blended Paste

**Figure 5:**
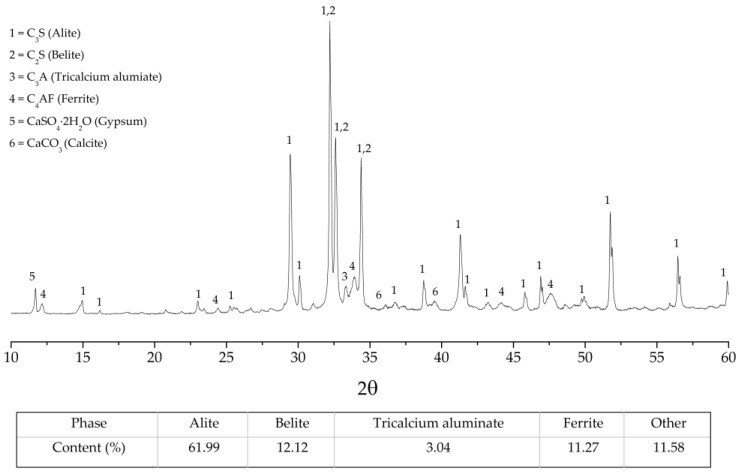
XRD patterns with phase composition of Ordinary Portland cement (OPC).

The CSA cement was mixed together to OPC with various fraction as listed in Table 4. The OPC phase composition, which obtained from the XRD patterns analysis through the Rietveld refinement technique, was shown in Figure 5. When compared to OPC cement, the incorporation of CSA cement leads to an increase in the amount of water required to produce pastes with a desired consistency (Table 4). This can be attributed to the higher amount of the H_2_O molecules needed for hydration of CSA cement when compared to the OPC hydrations. The setting times of cement paste rapidly shortened when the 20 wt.% of CSA cement was blended to the OPC cement. The initial and final setting times of OPC-CSA blend are gradually shortened by the increase of CSA cement fraction. These can be explained by the higher rate of hydrations of ye’elimite in CSA cement compared to hydration rate of OPC phases [31]. Moreover, the higher water demand during ettringite formation in CSA hydrations is associated with fewer H_2_O molecules and caused the setting times to be shorter [1].

The effect of CSA cement on the compressive strength of OPC is shown in Table 5. For 6 h curing period, the compressive strength of OPC paste could not be measured. For OPC-CSA blended, the compressive strength tended to increase with an increase of CSA cement fraction. The CSA40 shows more than 5 times compressive strength when compared to CSA20. These results suggest that the CSA cement is an appropriate admixture to improve early strength of OPC cement. The compressive strength of CSA-OPC blended are mostly higher than OPC paste until a 168 h curing period. For a longer curing period, the compressive strength of OPC paste is higher than that of all CSA-OPC blended samples. These results correspond to many previous investigations [28,40].

## 4. Conclusions

This study demonstrated the synthesis of calcium sulfoaluminate cement by using several waste materials as raw material. The chemical and mechanical properties of the raw materials, synthesized clinker, and hardened synthesized cement paste were studied in detail and the following conclusions were made according to the results of this paper:

- The particular industrial wastes show potential for application as raw material for CSA cement such as marble dust waste, flue gas desulfurization gypsum, and napier grass ash. CSA clinker with desired phase composition (i.e., C_2_S, C_4_A_3_$ and C_4_AF) can be successfully synthesized with the appropriate mixed proportion.
- CSA clinker fired at 1250 °C showed most similar phase content compared to designed composition. This clinker was used to study the hydrated pastes at various curing periods by using the FTIR technique.
- The replacement of OPC cement by CSA cement increased the water requirement for normal consistency, and shortened the initial and final setting times.
- Adding of the synthesized CSA cement to OPC cement is very helpful to improve compressive strength in the early age of hydration. However, the long-term compressive strength of synthesized CSA-OPC blended pastes were lower than that of the OPC paste.

## Figures and Tables

**Table 1:** Mix ratios of CSA/OPC blends for water requirement, setting times, and compressive strength tests.

Sample	OPC (wt.%)	CSA Clinker (wt.%)	Gypsum (wt.%)	CSA Cement (wt.%)
OPC	100	-	-	-
CSA20	80	16	4	20
CSA25	75	20	5	25
CSA30	70	24	6	30
CSA35	65	28	7	35
CSA40	60	32	8	40

**Table 2:** Chemical composition of the four industrial waste materials used, laboratory reagent grade Al_2_O_3_ powder, target chemical composition of the raw mixture, and measured chemical composition of the clinker fired at 1250 °C.

Raw Materials	Mix Proportions (wt.%.)	CaO	SiO_2_	Al_2_O_3_	Fe_2_O_3_	SO_3_	K_2_O	P_2_O_5_
MDW	41	100	-	-	-	-	-	-
NGA	10	3.58	69.92	8.42	7.74	-	7.23	1.18
CDW	10	-	68.65	15.62	5.37	-	9.62	-
FGDG	14	49.34	-	-	-	49.25	-	1.28
Al_2_O_3_	25	-	-	100	-	-	-	-
Target chemical composition of raw mixture		48.57	13.94	27.48	1.32	6.72	1.70	0.33
Measured chemical composition of calcined clinker at 1250 °C		49.13	14.20	24.83	2.14	7.01	0.43	0.09

**Table 3:** Target phase composition of CSA clinker phase and CSA clinker phase composition fired at various temperatures calculated by Rietveld refinement technique.

Phase	Target Phase Composition (wt.%.)	Clinker Phase Composition Fired at 1200 °C (wt.%.)	Clinker Phase Composition Fired at 1250 °C (wt.%.)	Clinker Phase Composition Fired at 1300 °C (wt.%.)
Ye’elimite (C_4_A_3_$)	50	46.25	48.11	47.41
β-Belite (C_2_S)	40	43.80	41.95	42.79
Brownmillerite (C_4_AF)	10	4.54	4.90	4.36
Mayenite		1.50	0.07	0.36
Bassanite		0.25	0.05	0.27
Good of fitness		3.26	3.25	3.24

**Table 4:** Water to binder ratios (*w*/*b*) to normal consistency and setting times of cement pastes.

Sample	% CSA	*w/b* to Normal Consistency	Initial Setting Time (minutes)	Final Setting Time (minutes)
OPC	0	0.272	116	195
CSA20	20	0.318	38	90
CSA25	25	0.328	28	60
CSA30	30	0.336	22	55
CSA35	35	0.369	17	35
CSA40	40	0.405	17	30

**Table 5:** Compressive strength of cement pastes at different curing periods.

Sample	% CSA	6 h (ksc)	12 h (ksc)	24 h (ksc)	72 h (ksc)	168 h (ksc)	336 h (ksc)	672 h (ksc)
OPC	0	-	171	413	519	697	817	850
CSA20	20	43	156	310	524	610	627	649
CSA25	25	54	212	434	476	549	594	600
CSA30	30	59	244	428	444	526	536	582
CSA35	35	101	274	439	442	456	481	493
CSA40	40	239	305	367	402	424	448	455
